# COVID-19 Epidemic: Clinical Characteristics of Patients in Pediatric Isolation Ward

**DOI:** 10.1177/0009922820941228

**Published:** 2020-07-09

**Authors:** Jian Zhu, Yabin Wu

**Affiliations:** 1 Department of Pediatric Respiration, Hubei Maternal and Child Health Care Hospital, Wuhan, China

**Keywords:** SARS-CoV-2, COVID-19, suspected COVID-19, isolation ward, clinical characteristics

## Abstract

In order to accurately admit children with COVID-19 to an isolation ward, our study retrospectively analyzed the clinical characteristics of children in isolation wards during the COVID-19 epidemic. It was found that 55 cases (83.3%) had fever and 48 cases (72.7%) coughed in the isolated area, 31 cases (47%) had a history of exposure, 26 cases (39.4%) had a decrease in lymphocytes (LYM), more than half had an increase in lactate dehydrogenase and creatine kinase isoenzyme, 14 cases (21.2%) had positive SARS-CoV-2 nucleic acid, 58 cases (87.9%) had abnormal chest computed tomography (CT), and 11 cases (16.7%) had sinus arrhythmia. Therefore, for some suspected children with COVID-19, we can make a comprehensive judgment through clinical symptoms, epidemiological history, LYM number, myocardial enzyme spectrum, chest CT, and electrocardiogram; put these children in an isolation ward for treatment; and then transfer them to a general ward for treatment after excluding COVID-19.

## Introduction

In January 2020, a new coronavirus was found in Wuhan, Hubei, China. The coronavirus research group of the International Committee for the Classification of viruses named it severe acute respiratory syndrome coronavirus 2 (SARS-CoV-2).^1^ The World Health Organization calls the pneumonia caused by the coronavirus COVID-19.^2^ Due to the characteristics of human-to-human transmission, COVID-19 has become popular all over the world and caused tens of thousands of deaths. Now it is a public health emergency of international concern over the global outbreak of novel coronavirus.^3^ At present, the National Health Commission of China has published the corresponding guidelines for diagnosis, treatment, prevention, and control of COVID-19.^4,5^ It is mentioned that medical institutions should set up isolation wards. The suspected or confirmed patients of COVID-19 were placed in the isolation ward first, and then pathogen or serological examination was performed. If evidence of COVID-19 infection is found, the patient will be transferred to a targeted treatment unit to continue treatment. At present, COVID-19 mainly infects adults, especially some elderly men.^6,7^ There are relatively few cases of infection in children. In this study, the clinical characteristics of children in isolation ward during the COIVID-19 epidemic were analyzed retrospectively, to provide basis for isolation and timely diagnosis of diseases in children. It is conducive to the scientific investigation of COVID-19 and provides an effective basis for COVID-19 prevention and control methods.

## Method

Because of the nature of this retrospective study, the Medical Ethics Committee of Hubei Maternal and Child Health Hospital approved the research plan and waived patient consent. The children in the pediatric isolation ward of our hospital from January 1, 2020, to February 29, 2020, were selected as the object of study. The basic data, epidemic history, symptoms, blood analysis, liver function, myocardial enzyme spectrum, chest computed tomography (CT) results, electrocardiogram (ECG) results, SARS-CoV-2 nucleic acid test results, hospitalization days, and hospitalization expenses of all the children in the isolation ward were collected.

Data processing was done by SPSS 25 statistical software. The counting data are described statistically by n (%). The measurement data of nonnormal distribution are described statistically by median and quartile [M(P_25_, P_75_)].

## Result

From January 1, 2020, to February 29, 2020, we collected data of 66 cases in the isolation ward. There were 37 males (56.1%) and 29 females (43.9%), and the male-to-female ratio was 1.28:1. There were 35 children without definite exposure history (53%). There were 17 children (25.8%) with suspected COVID-19 at home, and the children were in close contact with them. Only 14 patients (21.2%) had close contact with the confirmed COVID-19 patients. All the children in the isolation ward had different symptoms before admission. Among them, 55 cases (83.3%) had fever, 48 cases had cough (72.7%), 21 cases had stuffy nose (31.8%), and 24 cases had runny nose (36.4%; see Table 1).

**Table 1.** General Information of Children in Isolation Ward.

	Number of cases	Percentage (%)
Gender		
Male	37	56.1
Female	29	43.9
No exposure history	35	53.0
Contact with suspected cases	17	25.8
Contact with confirmed cases	14	21.2
Symptoms		
Fever	55	83.3
Cough	48	72.7
Stuffy nose	21	31.8
Runny nose	24	36.4

The age of the children ranged from 1 month to 13 years, with an average of 4.3 years. The number of children of school age was the largest, with 18 cases (27.3%). There were 17 cases (25.8%) of infancy. There were 14 cases of early childhood (21.2%), 11 cases of preschool age (16.6%), and 6 cases of puberty (9.1%; see Table 2).

**Table 2.** Age Distribution of Children in Isolation Ward.

Age stage	Number of cases	Percentage (%)
Infancy	17	25.8
Early childhood	14	21.2
Preschool age	11	16.6
School age	18	27.3
Puberty	6	9.1
Total	66	100

In the course of collecting case data, we found that not all children had data of the pathogen. Among them, the largest number of children were infected with *Mycoplasma pneumoniae*, 17 cases (25.8%), followed by SARS-CoV-2 infection, 14 cases (21.2%). There were 6 children with respiratory syncytial virus (9.1%). Only 2 cases (3%) were infected with Epstein-Barr virus (see Table 3).

**Table 3.** Children With a Clear Pathogen.

	Number of cases	Percentage (%)
*Mycoplasma pneumoniae*	17	25.8
Respiratory syncytial virus	6	9.1
Epstein-Barr virus	2	3
SARS-CoV-2	14	21.2

From the laboratory test data, we found that white blood cells level was normal in 44 cases (66.7%), increased in 15 cases (22.7%), and decreased in 7 cases (10.6%). The number of lymphocytes (LYM) was normal in 21 cases (31.8%), increased in 19 cases (28.8%), and decreased in 26 cases (39.4%). C-reactive protein level was increased in 23 cases (34.8%). In the liver function of children, alanine aminotransferase was increased in 11 cases (16.7%) and aspartate aminotransferase was increased in 25 cases (37.9%). In the myocardial enzymes, lactate dehydrogenase was increased in 37 cases (56.1%), CK-MB was increased in 36 cases (54.5%), and creatine kinase was increased in only 7 cases (10.6%). All the children in the isolation ward had tests for the detection of SARS-CoV-2 nucleic acid, and 14 of them were positive (21.2%; see Table 4).

**Table 4.** Laboratory Test Data of Children.

	Number of cases	Percentage (%)
WBC		
Increase	15	22.7
Normal	44	66.7
Decrease	7	10.6
LYM		
Increase	19	28.8
Normal	21	31.8
Decrease	26	39.4
CRP increase	23	34.8
ALT increase	11	16.7
AST increase	25	37.9
LDH increase	37	56.1
CK increase	7	10.6
CK-MB increase	36	54.5
SARS-CoV-2 nucleic acid positive	14	21.2

Abbreviations: WBC, white blood cell; LYM, lymphocytes; CRP, C-reactive protein; ALT, alanine aminotransferase; AST, aspartate aminotransferase; LDH, lactate dehydrogenase; CK, creatine kinase; CK-MB, creatine kinase isoenzyme.

All the children in the isolation ward had had chest CT at the time of admission. Among them, there were 8 cases without obvious abnormality (12.1%), 28 cases (42.4%) had speckled or patchy shadow, 6 cases (9.1%) had strip shadow, and 19 cases (28.8%) had ground glass opacity (GGO). A total of 4 cases (6.1%) were found to have atelectasis, and only 1 case (1.5%) had pleural effusion (see Table 5).

**Table 5.** Chest CT Results of Children.

	Number of cases	Percentage (%)
No obvious abnormality	8	12.1
Speckled or patchy	28	42.4
Stripe shape	6	9.1
GGO	19	28.8
Atelectasis	4	6.1
Pleural effusion	1	1.5

Abbreviations: CT, computed tomography; GGO, ground glass opacity.

Through the study of the ECG of the children, it was found that 27 cases showed sinus rhythm (40.9%) and 28 cases showed sinus tachycardia (42.4%), but we found that 11 cases showed sinus arrhythmia (16.7%; see Table 6).

**Table 6.** ECG Results of Children.

	Number of cases	Percentage (%)
Sinus rhythm	27	40.9
Sinus tachycardia	28	42.4
Sinus arrhythmia	11	16.7

Abbreviation: ECG, electrocardiogram.

Based on the statistical analysis of the hospitalization days of all the children in the isolation ward, it was found that the median and quartile of hospitalization days were 8 (7, 10) days, and the hospitalization cost was CNY 9269.32 (8168.2, 11204.33).

## Discussion

Coronavirus is a RNA virus that exists in nature. It can be divided into 4 subtypes: α, β, γ, and δ. SARS-CoV and Middle East respiratory syndrome coronavirus (MERS-CoV), which can cause serious respiratory diseases, belong to type β coronavirus.^8^ The genome sequence of SARS-CoV-2 was matched with that of type β coronavirus, but it was different from that of SARS-CoV and MERS-CoV. At present, some of the physical and chemical properties of SARS-CoV-2 refer to the studies of SARS-CoV and MERS-CoV.

At present, our diagnosis of COVID-19 is mainly based on the Diagnosis and Treatment of New Coronavirus Pneumonia (Trial Version 7) issued by the National Health Committee of the People’s Republic of China.^4^ It is mentioned that the diagnosis of suspected cases requires any one of the epidemiological history and conforms to any 2 of the clinical manifestations. If there is no clear history of epidemiology, it is necessary to comply with 3 of the clinical manifestations. Due to the age limitation, infections in most of the children are spread by family gatherings, and some of them have no obvious exposure history, which may lead to misdiagnosis or missed diagnosis, and finally bring difficulties to epidemic prevention and control.

Because COVID-19 is highly infectious, in order to screen suspected cases of COVID-19 and prevent the spread of the epidemic, the inpatient department has set up an isolation ward, which aims to make Infectious Diseases get early detection, early diagnosis, early isolation, and early treatment. Some patients with potential COVID-19 infection were further screened in the isolation ward in order to provide new clinical basis for the prevention and control of COVID-19 in the future.

In this study, by summarizing the clinical characteristics of children in the isolation ward, it was found that the male-to-female ratio was 1.28:1 in the isolation ward, and the percentage of male was higher than that of female. However, there is no sufficient evidence to prove that male children are more susceptible, which needs to be confirmed by further epidemiological investigation. There are 31 cases of children who had a history of exposure (47%), but only 14 cases (21.2%) were in close contact with COVID-19 patients. However, we also found that some children with COVID-19 had no clear history of exposure. It is suggested that COVID-19 cannot be excluded completely in children without definite epidemic history, and it may be judged comprehensively by combining other symptoms or examination results. Most of the symptoms in the isolation ward were fever and cough, and some children had stuffy nose and runny nose.^9^ But these symptoms have no obvious specificity compared with other pneumonia. At present, some of COVID-19 are asymptomatic infection,^10,11^ which suggests that it is not only from the clinical symptoms to judge whether the children should be admitted to the isolation ward. We found that the children in the isolation ward had not only SARS-CoV-2 infection but also *Mycoplasma pneumoniae* and respiratory syncytial virus infection. Previous studies have shown that the differential diagnosis of COVID-19 is *Mycoplasma pneumonia* and bronchiolitis.^12^ This is consistent with our findings.

The number of LYM decreased in 39.4% of the children in the isolation ward. This may be related to the fact that the virus inhibits LYM production or kills LYM.^13^ Lactate dehydrogenase and CK-MB were increased in more than half of the children, which may be caused by different degrees of myocardial cell damage caused by infection. This is similar to what Mugosa et al^14^ found in his study of influenza A (H1N1) virus. A total of 21.2% of the children in the isolation ward were SARS-CoV-2 nucleic acid positive, and the infection rate was high, which also suggested the necessity of screening children for COVID-19 in the isolation ward.

In the isolation ward, 87.9% of the patients had abnormal chest CT, including 28 cases with speckled or patchy shadow, 6 cases with stripe shape, and 19 cases with GGO. However, the current study found that the CT image of COVID-19 is complex and changeable, mainly manifested as multiple GGO of both lungs. This may be related to telangiectasia and hyperemia of alveolar septum, fluid exudation in alveolar cavity, and interstitial congestion of interlobular septum.^15^ This shows that the abnormality of chest CT is an important reference for children to enter the isolation ward.

Through the study of the ECG of the children, it was found that 16.7% of the ECG of the children showed sinus arrhythmia. This may also be related to cardiac hypoxia and cardiomyocyte damage caused by infection. This also suggests that we should pay attention to protect the heart function in the treatment of children in the isolation ward.

When the children were hospitalized in the isolation ward, we found that the hospitalization days were relatively long and the hospitalization costs were relatively high. This may be related to the need for multiple SARS-CoV-2 nucleic acid tests. Therefore, if we can rule out the risk of infectious diseases in the isolation ward as soon as possible, we can transfer the child to the general ward for further treatment, which can shorten the hospitalization time and treatment cost of the child.

Although nucleic acid detection is the gold standard for the diagnosis of COVID-19, due to the influence of many factors on the specimen, it is easy to have false negative.^16^ Therefore, we can comprehensively judge some suspected children with COVID-19 by clinical symptoms, history of epidemiology, number of LYM, myocardial zymogram, chest CT, and ECG. We can first put the suspected children with COVID-19 into the isolation ward for treatment, and then transfer them to the general ward for treatment after excluding COVID-19. In this way, it can play a certain role in the prevention and treatment of COVID-19. As this study belongs to a single-center study, and the sample size is small, there are some limitations, which may not reflect the actual situation of the disease. Therefore, further research is needed with a larger sample size and at multiple locations.

## Author Contributions

JZ contributed to acquisition, analysis, or interpretation of data, and drafted the manuscript. YW substantially contributed to conception or design and critically revised the manuscript for important intellectual content. All authors gave final approval, agree to be accountable for all aspects of the work in ensuring that questions relating to the accuracy or integrity of any part of the work are appropriately investigated and resolved.
